# A questionnaire-based survey on the epidemiological and clinical characteristics of SARS-CoV-2 infection in patients with chronic HBV infection and HIV infection

**DOI:** 10.3389/fpubh.2025.1536794

**Published:** 2025-03-13

**Authors:** Lu Chen, Chao Cai, Si-Jie Zheng, Liang Hong, Hui Zhao, Fei-Fei Su, Ming-Qin Lu

**Affiliations:** ^1^Department of Infectious Diseases, The First Affiliated Hospital of Wenzhou Medical University, Wenzhou, Zhejiang, China; ^2^Department of Infectious Diseases, The Third Affiliated Hospital of Wenzhou Medical University, Rui‘an People’s Hospital, Wenzhou, Zhejiang, China; ^3^Department of Infectious Diseases, Yueqing Affiliated Hospital of Wenzhou Medical University, Yueqing People’s Hospital, Wenzhou, Zhejiang, China; ^4^Department of Infectious Diseases, Wenzhou Central Hospital, The Sixth People’s Hospital of Wenzhou, Wenzhou, Zhejiang, China

**Keywords:** COVID-19, HBV, HIV, antiviral therapy, symptoms

## Abstract

**Background and aims:**

Traditional observational studies have yielded inconsistent findings regarding the association between COVID-19 and HBV/HIV infections, as well as the protective effects of antiviral therapy against severe COVID-19. This study aimed to investigate the potential links between the current use of antiviral therapy and the rates of severe acute respiratory syndrome coronavirus 2 (SARS-CoV-2) infection and symptoms of infection in patients with SARS-CoV-2 and HBV/HIV.

**Methods:**

Using a questionnaire-based survey, we recorded whether participants had been infected with SARS-CoV-2, and the symptoms and severity of COVID-19 after the illness.

**Results:**

Among 756 participants, chi-square tests showed a higher incidence of COVID-19 in the HBV infection group (75.6%, *p* = 0.047) and the HIV infection group (77.6%, *p* = 0.036). These two groups exhibited fewer symptoms than the control group (*p* < 0.001). The differences in the prevalence of most symptoms were also significant.

**Conclusion:**

Our findings suggest that patients with HBV or HIV infection have a higher risk of contracting SARS-CoV-2 than the general population; however, antiviral treatment relieves the symptoms of COVID-19.

## Introduction

Coronavirus disease 2019 (COVID-19) is an infectious disease caused by severe acute respiratory syndrome coronavirus 2 (SARS-CoV-2) (1) and first emerged in Wuhan City, China. The transmission of SARS-CoV-2 mainly occurs through respiratory secretions or droplets generated when people cough, sneeze, or exhale. Despite the many preventive measures taken by all countries worldwide, the organisms continue to spread rapidly worldwide, causing an ongoing pandemic. Globally, as of August 30, 2023, there have been 770,085,713 confirmed cases of COVID-19, including 6,956,173 deaths, making it a public health concern. To the best of our knowledge, most cases are mild or asymptomatic; however, a subset of patients with underlying conditions may develop severe respiratory problems, including pneumonia or acute respiratory distress, requiring mechanical assistance to breathe (2). Among those with symptoms, the most common presentations include fever, muscle aches, fatigue, and involvement of the upper and lower airways, resulting in nasal congestion, sore throat, anorexia, congestion, dry cough, and dyspnea. In addition to these respiratory symptoms, gastrointestinal, cardiovascular, and neurological manifestations have also been described (3).

China, with its large and increasingly aging population, faces a unique challenge due to the high prevalence of chronic diseases, including viral infections such as hepatitis B virus (HBV) and human immunodeficiency virus (HIV). Hepatitis B virus (HBV) infection is a major public health problem, with an estimated 70 million people infected. Chronic HBV infection can lead to liver cirrhosis, liver failure, and hepatocellular carcinoma, and it has been observed that COVID-19 patients with underlying HBV infection may experience more severe hepatic impairment (4, 5). Similarly, HIV infection is another significant health issue, with individuals living with HIV being at a higher risk of opportunistic infections due to compromised immune function. The immune dysregulation associated with HIV infection may further exacerbate the severity of COVID-19. However, effective antiretroviral therapy (ART) has been shown to partially restore immune function in HIV patients.

Primary treatments for COVID-19 include antiviral drugs, immunomodulators, neutralizing antibodies, and cell and gene therapies (6). Oral drugs to suppress SARS-CoV-2 have been developed and are being marketed. Antiviral agents against COVID-19 include angiotensin-converting enzyme 2 inhibitors, membrane fusion inhibitors, RNA polymerase inhibitors, and 3CL protease inhibitors (7). Similar agents are widely used to treat HBV or HIV infections. For example, atazanavir is an antiretroviral protease inhibitor that is primarily used to treat HIV. If administered intravenously, it can reach the lungs and help cure pulmonary fibrosis (8). Other multitarget drugs for COVID-19 therapy include anticoagulants and antiviral drugs commonly used to treat hepatitis C virus (HCV), HBV (entecavir), HSV (penciclovir), and CMV.

However, whether antiviral therapy can protect HIV/HBV-infected individuals from severe COVID-19 remains a matter of debate. Given these reports, we hypothesized that the current use of antiviral agents in patients with HBV or HIV infection could affect their susceptibility to SARS-CoV-2 infection and its symptoms. Using a questionnaire survey, we investigated the potential links between the current use of antiviral therapy and the rates of SARS-CoV-2 infection and symptoms of infection in patients with SARS-CoV-2.

## Methods

### Study design and population

Our study included 756 participants, who were divided into three groups based on their infection status: HBV infection group (*n* = 357), HIV infection group (*n* = 170), and control group (*n* = 229). Patients with HBV or HIV infected were from The First Affiliated Hospital of Wenzhou Medical University, Wenzhou Sixth People’s Hospital, Rui’an People’s Hospital, and Yueqing People’s Hospital (Wenzhou, Zhejiang, China). Participants in control group were caregivers of both inpatients and outpatients. COVID-19 diagnosis methods include: getting a nucleic acid test at the hospital, self-testing for antigens at home, or experiencing COVID-19 symptoms after close contact with a confirmed COVID-19 patient. Individuals were asked to complete a survey questionnaire during face-to-face interviews. The responses were recorded in an Excel spreadsheet. No age or sex restrictions were imposed. All procedures were performed in accordance with the guidelines of the institutional ethics committee and adhered to the tenets of the Declaration of Helsinki. Participants’ information remained anonymous throughout the study. This study was exempt from informed consent and was approved by the Ethics Committee of the First Affiliated Hospital of Wenzhou Medical University (approval number: KY2023-R183) in China.

### Questionnaire

The questionnaire included basic demographic information such as age, sex, height, weight, and underlying medical conditions. Participants were asked whether they had suffered from COVID-19 and the main symptoms they had experienced during SARS-CoV-2 infection. A list of 11 major symptoms was provided to the respondents, and were asked to mark the symptoms they had experienced. The symptoms included fever, cough, diarrhea, muscle aches, fatigue, sore throat, difficulty breathing, chest pain or pressure, headache, nasal congestion, and runny nose. The respondents were also asked if they had experienced any other symptoms. These key signs and symptoms of SARS-CoV-2 infection were selected based on a literature review and the experience of local clinicians.

### Statistical analyses of the data

For continuous variables, data are expressed as mean ± standard deviation (SD) (normal distribution) or median (interquartile range, IQR) for non-normal distribution. Categorical variables are presented as numbers (%). The demographic characteristics of the participants in the different groups were compared using the Mann–Whitney U test and chi-square test. Statistical significance was set at *p* < 0.05.

## Results

### General characteristics of the study population

Our study included 756 respondents. The participants’ general characteristics are listed in Table 1. The median age was 44.19 years, and 462 (61.11%) were men with a median BMI of 22.78. A total of 680 participants were vaccinated. Among them, 440, 192, 19, and 29 participants had completed three, two, one, and four doses, respectively. Overall, 159 participants had a history of smoking, and 357 and 170 were infected with HBV and HIV, respectively. In this study, 558 participants had been diagnosed with COVID-19. Fever, cough, and fatigue were the primary symptoms. Other symptoms included headache, myalgia, nasal obstruction, and runny nose.

**Table 1:** General characteristics of all the participants (*N* = 756).

Sex (male), *n* (%)	462 (61.11)
Age (in years)	44.19 ± 12.00
BMI	22.78 ± 2.99
Vaccination	
Unvaccinated	76 (10.05)
1 dose	19 (2.51)
2 doses	192 (25.40)
3 doses	440 (58.20)
4 doses	29 (3.84)
Comorbidities, *n* (%)	
Smoking	159 (21.03)
Hypertension	95 (12.57)
Diabetes	26 (3.44)
COVID-19 infection	558 (73.81)

BMI, body mass index.

### Comparison of prevalence of COVID-19 and symptoms after illness

The general characteristics of the 357 patients with HBV infection (the HBV infection group) are shown in Table 2. The proportion of males in the HBV infection group (225, 63.0%) was higher than that in the control group (92, 40.2%). Vaccination rates also differed between the two groups. The number of individuals who had never been vaccinated (41, 11.49%) was higher in the HBV group. Chi-square tests were performed after stratification for sex and vaccination status to eliminate the effects of these two factors. The Breslow-Day test for homogeneity of odds ratios showed *p* > 0.05, indicating that there was no confounding effect among the confounders. We then compared the prevalence of COVID-19 between the HBV infection group and the control group. The incidence of COVID-19 was significantly higher in the HBV infection group (75.6%, *n* = 270) compared to the control group (*p* = 0.047). Many individuals presented with up to 12 symptoms, while others were asymptomatic. We compared the average number of symptoms between the HBV-infected and control groups to determine disease severity. The HBV infection group exhibited significantly fewer symptoms than the control group (*p* < 0.001). Furthermore, the difference in the prevalence of most symptoms between the HBV infection and control groups was significant, as determined by the chi-square test. In addition, the maximum temperature during fever in patients in the HBV infection group was lower than that in the control group (*p* < 0.05). Most people had a fever for less than 3 days. The duration of cough in the HBV infection group was shorter (*p* < 0.001). Based on the above results, we can conclude that the symptoms of COVID-19 in patients with HBV infection were relatively mild (Table 3).

**Table 2:** Comparison of COVID-19 prevalence between HBV infection and control groups.

	Control (*N* = 229)	HBV infection (*N* = 357)	*p-*value
Male, *n* (%)	92 (40.2)	225 (63.0)	<0.001
Age (in years), Median (IQR)	43 (18)	44 (16)	0.410
BMI, Median (IQR)	22.43 (4.18)	22.73 (3.73)	0.099
Vaccination, *n* (%)			<0.001
Unvaccinated	7 (3.06)	41 (11.49)	
1 dose	5 (2.18)	6 (1.68)	
2 doses	43 (18.77)	88 (24.65)	
3 doses	159 (69.43)	213 (59.66)	
4 doses	15 (6.55)	9 (2.52)	
Comorbidities			
Smoking, *n* (%)	27 (11.8)	62 (17.37)	0.66
Hypertension, *n* (%)	24 (10.5)	38 (10.64)	0.95
Diabetes, *n* (%)	8 (3.5)	9 (2.52)	0.494
COVID-19 infection, *n* (%)	156 (68.1)	270 (75.6)	0.047

BMI, body mass index; IQR, interquartile range.

**Table 3:** Comparison of post-illness symptoms of COVID-19 between the HBV infection and control groups.

	Control (*N* = 156)	HBV infection (*N* = 270)	*p-*value
Male, *n* (%)	65 (41.67)	167 (61.85)	<0.001
Age (in years), Median (IQR)	43 (18)	44 (17)	0.472
BMI, Median (IQR)	22.811 ± 3.097	23.083 ± 2.634	0.337
Vaccination, *n* (%)			<0.001
Unvaccinated	2 (1.28)	30 (11.1)	
1 dose	2 (1.28)	5 (1.85)	
2 doses	31 (19.87)	69 (2.56)	
3 doses	112 (71.79)	162 (60)	
4 doses	9 (5.77)	4 (1.48)	
Comorbidities			
Smoking, *n* (%)	15 (9.62)	43 (15.93)	0.067
Hypertension, *n* (%)	18 (11.54)	31 (11.48)	0.986
Diabetes, *n* (%)	5 (3.21)	5 (1.85)	0.374
Number of symptoms after illness, Median (IQR)	4 (3.5)	3 (3)	<0.001
Fever, *n* (%)	135 (86.54)	203 (75.19)	0.005
Maximum temperature (°C)			
37.6–38, *n* (%)	15 (11.1)	32 (15.76)	<0.001
38.1–38.5, *n* (%)	34 (25.19)	75 (36.95)	
38.6–39, *n* (%)	35 (25.93)	56 (27.59)	
39.1–39.5, n (%)	33 (24.44)	27 (13.3)	
39.6–40, *n* (%)	15 (11.1)	10 (4.93)	
Above 40, *n* (%)	3 (2.22)	3 (1.48)	
Duration			
1–3 days, *n* (%)	110 (81.48)	158 (77.83)	0.460
3–7 days, *n* (%)	22 (16.30)	43 (21.18)	
7–14 days, *n* (%)	3 (2.22)	2 (0.97)	
More than 14 days, *n* (%)	0 (0)	0 (0)	
Cough and expectoration, *n* (%)	106 (67.95)	165 (61.11)	0.158
Duration			
1–3 days, *n* (%)	17 (16.04)	72 (43.64)	<0.001
3–7 days, *n* (%)	33 (31.13)	37 (22.42)	
1–2 weeks, *n* (%)	23 (21.70)	27 (16.36)	
More than 2 weeks, *n* (%)	33 (31.13)	29 (17.58)	
Cough and expectoration, *n* (%)			
Muscle aches, *n* (%)	88 (56.41)	93 (34.44)	<0.001
Fatigue, *n* (%)	93 (59.62)	116 (42.96)	0.001
Headache, *n* (%)	59 (37.82)	54 (20)	<0.001
Sour throat, *n* (%)	50 (32.05)	45 (16.67)	<0.001
Runny nose, *n* (%)	50 (32.05)	42 (15.56)	<0.001

BMI, body mass index; IQR, interquartile range.

**Figure 1:**
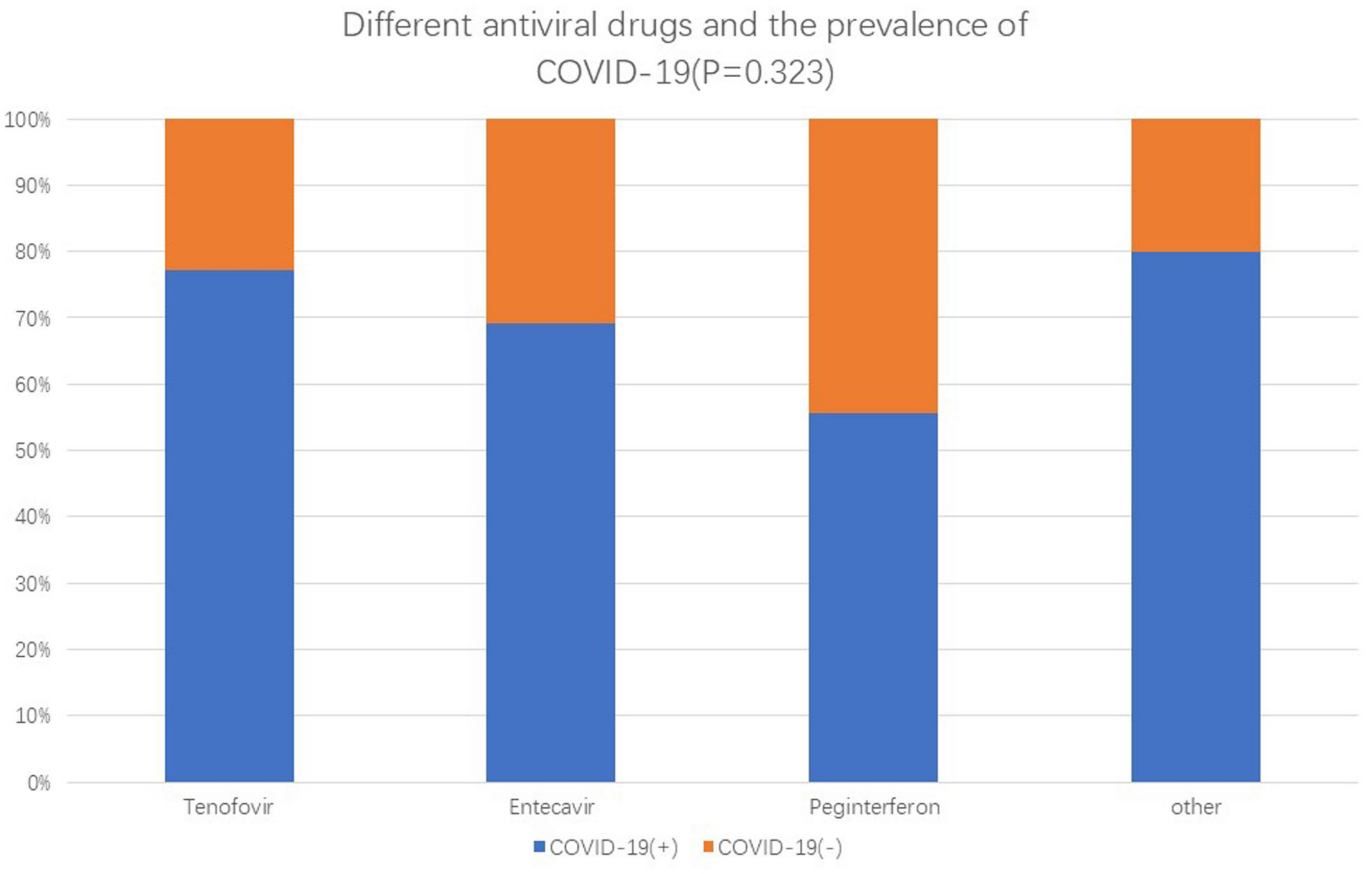
Prevalence of antiviral drugs and COVID-19.

We further investigated the factors associated with mild COVID-19 symptoms in patients with HBV. Of the 357 patients with HBV, 265 received antiviral treatment and 92 were never treated. We compared the prevalence and symptoms of COVID-19 between these two groups. A lower prevalence of SARS-CoV-2 infection and milder symptoms were observed in patients receiving antiviral treatment (Tables 4, 5). Therefore, it can be concluded that antiviral treatment is related to the severity of COVID-19 symptoms. We examined the relationship between different antiviral drugs and the prevalence of COVID-19. However, there were no significant differences between the groups (Figure 1).

**Table 4:** Comparison of COVID-19 prevalence between the treated and untreated groups.

	Treated (*N* = 265)	Untreated (*N* = 92)	*p-*value
Age (in years), Median (IQR)	44 (17.5)	44.5 (15)	0.295
Male, *n* (%)	174 (65.67)	51 (55.43)	0.08
BMI, Median (IQR)	22.86 (3.67)	22.82 (3.71)	0.283
Vaccination			
Unvaccinated, *n* (%)	28 (10.57)	13 (14.13)	0.554
1 dose, *n* (%)	4 (1.51)	2 (2.17)	
2 doses, *n* (%)	62 (23.40)	26 (28.26)	
3 doses, *n* (%)	165 (62.26)	48 (52.17)	
4 doses, *n* (%)	6 (2.26)	3 (3.26)	
COVID-19 infection (*n*, %)	194 (73.2)	76 (82.6)	0.07

BMI, body mass index; IQR, interquartile range.

**Table 5:** Comparison of symptoms after illness between the treated and untreated groups.

	Treated (*N* = 194)	Untreated (*N* = 76)	
Age (in years), Median (IQR)	44 (18)	44.5 (15)	0.214
Male, *n* (%)	124 (63.92)	43 (56.58)	0.264
BMI, Median (IQR)	23.34 ± 2.72	22.79 ± 2.77	0.394
Vaccination			0.258
Unvaccinated, *n* (%)	17 (8.76)	13 (17.11)	
1 dose, *n* (%)	3 (1.55)	2 (2.63)	
2 doses, *n* (%)	48 (24.74)	21 (27.63)	
3 doses, *n* (%)	123 (63.40)	39 (51.32)	
4 doses, *n* (%)	3 (1.55)	1 (1.32)	
Number of symptoms after illness, Median (IQR)	2 (2)	3 (3)	<0.001
Fever, *n* (%)	141 (72.68)	38 (50)	<0.001
Maximum temperature (°C)			
37.6–38, *n* (%)	27 (19.15)	3 (7.89)	0.017
38.1–38.5, *n* (%)	58 (41.13)	13 (34.21)	
38.6–39, *n* (%)	35 (24.82)	11 (28.95)	
39.1–39.5, *n* (%)	14 (9.93)	8 (21.05)	
39.6–40, *n* (%)	5 (3.55)	2 (5.26)	
Above 40, *n* (%)	2 (1.42)	1 (2.63)	
Duration			
1–3 days, *n* (%)	117 (82.98)	29 (76.32)	0.288
3–7 days, *n* (%)	24 (17.02)	7 (18.42)	
7–14 days, *n* (%)	0 (0)	2 (5.26)	
More than 14 days, *n* (%)	0 (0)	0 (0)	
Cough and expectoration, *n* (%)	88 (45.36)	51 (67.11)	0.001
Duration			
1–3 days, *n* (%)	49 (55.69)	20 (39.22)	0.003
3–7 days, *n* (%)	22 (25)	5 (9.8)	
1–2 weeks, *n* (%)	9 (10.23)	12 (23.53)	
More than 2 weeks, *n* (%)	8 (9.09)	14 (27.45)	
Muscle aches, *n* (%)	57 (29.38)	36 (47.37)	0.005
Fatigue, *n* (%)	72 (37.11)	44 (57.89)	0.002
Headache, *n* (%)	38 (19.59)	16 (21.05)	0.787
Sour throat, *n* (%)	23 (11.86)	22 (28.95)	0.001
Runny nose, *n* (%)	23 (11.86)	4 (5.26)	0.104

BMI, body mass index; IQR, interquartile range.

The prevalence of COVID-19 in the HIV infection group and control group was also investigated (Table 6). The number of patients with SARS-CoV-2 infection was significantly higher in the HIV infection group (132, 77.6%) than in the control group (156, 68.1%). The total number of HIV-positive individuals with SARS-CoV-2 infection was 132, including two asymptomatic patients. All patients with HIV received antiviral therapies. Fever was observed in 72.7% (*n* = 96/132) of the patients, 50% (*n* = 66/132) had cough, and 26.5% (*n* = 35/132) reported muscle aches. Sore throat and headache were experienced by 16.7% (*n* = 22/132) and 66.8% (*n* = 99/132) of the patients, respectively. The frequency of these symptoms was lower in the HIV infection group than in the control group (all *p* < 0.05). In the present study, individuals living with HIV who were also diagnosed with COVID-19 displayed less severe symptoms.

**Table 6:** Comparison of the prevalence and post-illness symptoms of COVID-19 between the HIV-infected and control groups.

	HIV infection (*N* = 170)	Control group (*N* = 229)	*p-*value
Male, *n* (%)	145, 85.29%	92 (40.2)	<0.001
Age (in years), Median (IQR)	43 (19)	43 (18)	0.544
BMI, Median (IQR)	22.49 (4.26)	22.43 (4.18)	0.654
Vaccination, *n* (%)			
Unvaccinated	28 (16.47)	7 (3.06)	<0.001
1 dose	8 (4.71)	5 (2.18)	
2 doses	61 (35.88)	43 (18.77)	
3 doses	68 (40)	159 (69.43)	
4 doses	5 (2.94)	15 (6.55)	
Comorbidities			
Smoking, *n* (%)	70	27 (11.8)	<0.001
Hypertension, *n* (%)	33 (19.4)	24 (10.5)	0.012
Diabetes, *n* (%)	9 (5.3)	8 (3.5)	0.379
COVID-19 infection, *n* (%)	132 (77.6)	156 (68.1)	0.036

	HIV infection (*N* = 132)	Control group (*N* = 156)	*p-*value
Male, *n* (%)	112 (84.85)	65 (41.67)	<0.001
Age (in years), Median (IQR)	41.5 (19)	43 (18)	0.359
BMI, Median (IQR)	22.54 ± 3.25	22.81 ± 3.10	0.467
Vaccination, *n* (%)			<0.001
Unvaccinated	18 (13.64)	2 (1.28)	
1 dose	5 (3.79)	2 (1.28)	
2 doses	55 (41.67)	31 (19.87)	
3 doses	50 (37.87)	112 (71.79)	
4 doses	4 (3.03)	9 (5.77)	
Number of symptoms after illness, Median (IQR)	2 (2)	4 (3.5)	<0.001
Fever, *n* (%)	96 (72.7)	135 (86.5)	0.003
Maximum temperature (°C)			
37.6–38, *n* (%)	58 (60.42)	15 (11.1)	<0.001
38.1–38.5, *n* (%)	21 (21.88)	34 (25.19)	
38.6–39, *n* (%)	11 (11.46)	35 (25.93)	
39.1–39.5, *n* (%)	4 (4.17)	33 (24.44)	
39.6–40, *n* (%)	2 (2.08)	15 (11.1)	
Above 40, *n* (%)	0 (0)	3 (2.22)	
Duration			
1–3 days, *n* (%)	84 (87.5)	110 (81.48)	0.200
3–7 days, *n* (%)	12 (12.5)	22 (16.30)	
7–14 days, *n* (%)	0 (0)	3 (2.22)	
More than 14 days, *n* (%)	0 (0)	0 (0)	
Cough and expectoration, *n* (%)	66 (50)	106 (67.95)	0.002
Duration			
1–3 days, *n* (%)	6 (9.09)	17 (16.04)	0.002
3–7 days, *n* (%)	12 (18.18)	33 (31.13)	
1–2 weeks, *n* (%)	11 (16.67)	23 (21.70)	
More than 2 weeks, *n* (%)	37 (56.06)	33 (31.13)	
Muscle aches, *n* (%)	35 (26.5)	88 (56.4)	<0.001
Fatigue, *n* (%)	40 (30.3)	93 (59.6)	<0.001
Sour throat, *n* (%)	22 (16.7)	50 (32.1)	0.003
Runny nose, *n* (%)	22 (16.7)	50 (32.1)	0.003
Headache, *n* (%)	9 (6.8)	59 (37.8)	<0.001

BMI, body mass index; IQR, interquartile range.

## Discussion

In this study, we aimed to explore the potential association between underlying HBV or HIV infection, antiviral treatment, and SARS-CoV-2 infection susceptibility in 756 participants. In addition, we evaluated whether the symptoms of COVID-19 were influenced by HBV or HIV treatment. Our findings indicate that patients with HBV or HIV infections are at a higher risk of contracting SARS-CoV-2 compared to the general population. However, antiviral treatment appears to mitigate the severity of COVID-19 symptoms.

Numerous previous studies have investigated the association between prior HBV and SARS-CoV-2 infection. In a group of 326 patients with confirmed COVID-19, Chen et al. explored the clinical features of individuals with SARS-CoV-2/HBV co-infection and established that co-infection with HBV had a mild effect on liver function, did not affect the COVID-19 outcome, and did not affect the length of hospital stay (9). A systematic review and meta-analysis investigated the impact of HBV coinfection on hospitalized COVID-19 patients, finding no significant increase in mortality or ICU admission rates (10). A territory-wide retrospective cohort study conducted throughout Hong Kong including 5,639 patients (353 and 359 patients with current and past HBV infection, respectively) reported no association between current or past HBV infection and increased liver injury or mortality in COVID-19 (11). He et.al investigated the impact of SARS-CoV-2 and HBV coinfection on COVID-19 outcomes, finding that while coinfection does not significantly worsen COVID-19 severity, it is associated with more severe immune and hepatic disturbances at disease onset, necessitating careful management of coinfected patients (12). However, other studies reported conflicting results. Yang et al. found that patients co-infected with SARS-CoV-2 and HBV at the HBeAg (+) CHB/infection stage had an elevated risk of poor prognosis (13). Abnormal liver function partially mediated the increased risk of co-infection. Another meta-analysis indicated that COVID-19 patients with HBV had a higher risk of mortality (OR = 1.65, I2 = 58, and 95% CI 1.08–2.53) and severity (OR = 1.90, I2 = 44, and 95% CI 1.62–2.24) than those without HBV infection (14).

Our study revealed that the prevalence of COVID-19 was higher among patients with HBV and HIV infections (*p* < 0.05). Several studies have attempted to elucidate the reason for this correlation. HBV infection can elicit various immunomodulatory effects, resulting in weak or absent virus-specific T-cell reactivity. T-cells are exhausted during HBV infection and are gradually unable to clear the virus, losing both effector function and memory T-cell characteristics before complete deletion. This process is commonly referred to as T-cell exhaustion (TEX) (15) and puts patients with HBV at a higher risk of SARS-CoV-2-induced disease owing to compromised immunity. These findings indicate that the immune status of the host, to a certain extent, could have an impact on the outcomes of SARS-CoV-2 infection.

However, no studies have focused on the correlation between illness severity and symptoms. In our study, we were surprised to find that patients with CHB had milder symptoms after COVID-19. In our study, most patients with HBV received antiviral therapy. Therefore, we examined the association between the baseline use of antivirals and the severity of COVID-19. People who had been treated with antiviral drugs had milder symptoms of COVID-19. We determined the possible reasons for this through an extensive literature review. Studies have reported that tenofovir antiviral therapy may be associated with COVID-19 prognosis (16). Research has shown that the phosphorylated forms of both tenofovir disoproxil fumarate and tenofovir alafenamide have *in vitro* activity against the SARS-CoV-2 RNA-dependent RNA polymerase (RdRp) (17). A recent large-scale cohort study conducted in Spain found that patients with CHB treated with tenofovir had reduced incidence of SARS-CoV-2 infection (0.4%), indirectly reflecting the beneficial effects of tenofovir on the resistance to SARS-CoV-2 (18). The coronavirus RdRp is a well-established drug target. Nucleotide analogs that inhibit polymerases constitute an important class of antiviral agents. Mohamed highlighted a drug repurposing system, noting that antivirals against HBV, HCV, or HIV could be evaluated as COVID-19 therapeutics (8). Therefore, antiviral treatment can influence the severity of COVID-19 symptoms.

Several studies have also been conducted on patients with HIV and COVID-19. One potential cohort study comprising 5,683 patients with HIV observed a decreased incidence of COVID-19 in patients with HIV compared to the general population (19). However, the study was limited because it did not regulate use of antiviral treatment. Several other prospective cohort studies with limited study sizes have demonstrated comparable rates of SARS-CoV-2 infection in patients with HIV and the general population. A San Francisco-based study of 4,252 patients with HIV suggested that these patients were more vulnerable to SARS-CoV-2 infection than those without HIV (20, 21). A systematic review and meta-analysis concluded that while HIV infection increases the risk of hospital admission for COVID-19 patients, it does not significantly impact the severity of COVID-19 or mortality in unadjusted analyses, although adjusted analyses suggest a potential link to increased mortality based on limited data (22). Many experts initially considered people living with HIV as a vulnerable group with respect to SARS-CoV-2 infection because of the greater burden of some comorbidities, higher systemic inflammation, and the presence of some degree of immune alteration, even among those receiving effective antiviral therapy with immune reconstitution. However, it remains unclear whether this is true (23–25).

Our findings are consistent with previous studies, indicating that although the infection rate of COVID-19 is higher among HIV-infected individuals, the severity of symptoms is not necessarily greater. This may be attributed to the widespread use of antiviral therapies among HIV patients, which may help to mitigate the symptoms of COVID-19. Moreover, individuals living with HIV may be more vigilant about health management and protective measures in their daily lives, which could also contribute to the milder symptoms observed. However, despite the milder symptoms, HIV-infected individuals should remain cautious and continue to adhere to public health recommendations to reduce the risk of infection and potential severe outcomes.

In summary, our study showed higher COVID-19 infection rates among HBV and HIV patients, which can be attributed to several factors: (1) Weakentem: Both HBV and HIV infections compromise the immune system, making patients more susceptible to SARS-CoV-2. (2) Chronic Inflammation: Chronic viral infections often lead to a heightened inflammatory state, which may facilitate SARS-CoV-2 entry and replication. (3) Overlap in Risk Factors: HBV and HIV patients often share common risk factors with COVID-19, such as older age and comorbidities. (4) Healthcare Utilization: Frequent healthcare visits may expose these patients to a higher risk of SARS-CoV-2 infection. Nevertheless, we observed a significant association between antiviral therapy and milder symptoms of COVID-19. This phenomenon may be achieved through several mechanisms. Firstly, antiviral treatments (such as nucleoside analogs and protease inhibitors used for HBV and HIV) may have a direct antiviral effect on SARS-CoV-2 by inhibiting the virus’s RNA-dependent RNA polymerase (RdRp), thereby reducing viral replication. Secondly, antiviral therapy helps regulate the immune system by lowering viral load, which reduces chronic inflammation and immune activation, creating a more favorable environment for the body to combat SARS-CoV-2. Additionally, effective antiviral treatment can decrease the risk of opportunistic infections, indirectly reducing the severity of COVID-19. Lastly, antiviral therapy may enhance immune surveillance, enabling the body to mount a stronger and more timely response to SARS-CoV-2 infection. These mechanisms collectively may explain why patients receiving antiviral therapy experience milder symptoms after contracting COVID-19. Future research will further explore these potential mechanisms to better understand the role of antiviral therapy in mitigating the severity of COVID-19. This study has several limitations and the results must be interpreted with caution. All individuals participated in the study using a questionnaire. Although we used face-to-face questions and answers, it still had a certain subjectivity. Another limitation of this study was that we could not guarantee that most of the included population had been exposed to SARS-CoV-2. There are several potential confounders, including individual hygiene (i.e., hand washing), social distancing, and mask-wearing, which may have influenced our results. In addition, this cohort lacked detailed history and laboratory information on chronic hepatitis B disease status (e.g., HBV DNA levels, stages of liver fibrosis or whether they have AIDS in HIV-infected patients). Finally, this study was based solely on a questionnaire and therefore, warrants further investigation using randomized controlled trials to evaluate the effects of antiviral agents on the treatment and prevention of COVID-19. Despite its limitations, to the best of our knowledge, this is the first questionnaire-based study to investigate the association between COVID-19 risk and both underlying HBV or HIV infection and the use of antiviral therapeutics.

## Conclusion

Our findings suggest that patients with HBV or HIV have a higher risk of contracting SARS-CoV-2 than the general population; however, antiviral treatment relieves the symptoms of COVID-19. More samples are needed to study the effect of the type of antiviral drug and the duration of its use.

## Data Availability

The original contributions presented in the study are included in the article/supplementary material, further inquiries can be directed to the corresponding authors.
